# Winners vs. losers: *Schistosoma mansoni* intestinal and liver eggs exhibit striking differences in gene expression and immunogenicity

**DOI:** 10.1371/journal.ppat.1012268

**Published:** 2024-05-30

**Authors:** Kristýna Peterková, Lukáš Konečný, Tomáš Macháček, Lucie Jedličková, Franziska Winkelmann, Martina Sombetzki, Jan Dvořák

**Affiliations:** 1 Department of Parasitology, Faculty of Science, Charles University, Prague, Czechia; 2 Department of Zoology and Fisheries, Center of Infectious Animal Diseases, Faculty of Agrobiology, Food and Natural Resources, Czech University of Life Sciences, Prague, Czechia; 3 Department of Ecology, Center of Infectious Animal Diseases, Faculty of Environmental Sciences, Czech University of Life Sciences, Prague, Czechia; 4 Universitätsmedizin Rostock, Zentrum für Innere Medizin, Abteilung für Tropenmedizin, Infektionskrankheiten und Sektion Nephrologie, Rostock, Germany; 5 Institute of Organic Chemistry and Biochemistry, Czech Academy of Sciences, Prague, Czechia; Texas Biomedical Research Institute, UNITED STATES

## Abstract

The eggs of the blood fluke *Schistosoma mansoni* are the main cause of the clinical manifestations of chronic schistosomiasis. After laying, the egg “winners” attach to the endothelium of the mesenteric vein and, after a period of development, induce the growth of a small granuloma, which facilitates their passage to the intestinal lumen. Egg “losers” carried by the bloodstream to non-specific tissues also undergo full development and induce large granuloma formation, but their life ends there. Although these trapped eggs represent a dead end in the parasite life cycle, the vast majority of studies attempting to describe the biology of the *S*. *mansoni* eggs have studied these liver-trapped “losers” instead of migrating intestinal “winners”. This raises the fundamental question of how these eggs differ. With robust comparative transcriptomic analysis performed on *S*. *mansoni* eggs isolated 7 weeks post infection, we show that gene expression is critically dependent on tissue localization, both in the early and late stages of development. While mitochondrial genes and venom allergen-like proteins are significantly upregulated in mature intestinal eggs, well-described egg immunomodulators IPSE/alpha-1 and omega-1, together with micro-exon genes, are predominantly expressed in liver eggs. In addition, several proteases and protease inhibitors previously implicated in egg-host interactions display clear tissue-specific gene expression patterns. These major differences in gene expression could be then reflected in the observed different ability of liver and intestinal soluble egg antigens to elicit host immune responses and in the shorter viability of miracidia hatched from liver eggs. Our comparative analysis provides a new perspective on the biology of parasite’s eggs in the context of their development and tissue localization. These findings could contribute to a broader and more accurate understanding of parasite eggs interactions with the host, which have historically been often restricted to liver eggs and sometimes inaccurately generalized.

## Author summary

The egg of *Schistosoma mansoni* is the main causative agent of pathologies of chronic schistosomiasis, a neglected tropical disease with a significant global health burden. Pathology is primarily caused by a high number of eggs being lodged in host organs over time, causing inflammation and tissue damage. While trapped “loser” eggs causing the disease have been extensively studied, little attention has been paid to the successful “winner” eggs that pass through the intestinal wall and are responsible for the continuation of the life cycle. Here we provide a comprehensive analysis of the gene expression profiles of the parasite’s eggs based on their maturity and, most importantly, their tissue localization. We revealed that eggs differ substantially based on their tissue localization from an early development. In addition to genes associated with development and metabolism, we have described differential expression of known immunomodulators, which may be reflected by the differential capacity of eggs to elicit a host immune response. Furthermore, we demonstrate that genes encoding proteins potentially involved in host-parasite interactions exhibit clear tissue-dependent expression patterns. Our research provides novel insights into the biology of *S*. *mansoni* eggs and provides a robust data source for new avenues of investigation related to this important pathogen.

## 1 Introduction

The eggs of blood fluke *Schistosoma mansoni* are the main causative agent of the clinical manifestations of chronic schistosomiasis. In the blood vessels of a definitive vertebrate host, adult schistosomes form pairs, with the female laying up to 300 eggs per day directly into the bloodstream [1]. After oviposition, the eggs develop in the host for approximately 6 days [2]. During this time, they increase in volume and undergo embryonic development, leading to the formation of miracidium larvae [3,4]. As they develop, they induce the formation of small tissue granulomas, which later facilitate their passage from the mesenteric veins through the intestinal wall to the intestinal lumen [5]. Once there, they are shed with the host’s feces into the external environment [6].

However, between one third to half of laid eggs are carried by the bloodstream to non-specific tissues, primarily the liver, where they remain trapped [1,7]. These eggs also undergo full development up to the formation of viable miracidia, but their life ends in these tissues [8]. It is important to note that it is primarily these trapped "losers" that are responsible for chronic pathologies as they trigger strong immune responses leading to the formation of large tissue granulomas. This process results in extensive fibrosis, organomegaly, and tissue damage [9]. Understanding the mechanisms underlying these pathologies of schistosomiasis is critical for developing effective treatment strategies, and for this reason, the liver-trapped eggs have historically received much attention. It has been over 20 years since excretory-secretory products (ESPs) of schistosome eggs were collected and partially characterized for the first time using liver-derived eggs [3]. Since then, the composition of these ESPs has been described in detail several times [10–12]. Certain key molecules that induce granuloma formation have been identified, as well as molecules that modulate the host immune response and regulate inflammation [13–18].

With the increasing amount of transcriptomic data from different developmental stages of *S*. *mansoni*, it also became apparent that the eggs have a unique gene expression profile [19]. In the most recent transcriptomic studies, egg-specific genes responsible for embryonic and miracidial development were identified, alongside a distinct set of genes associated with modulation of immunity and other physiological processes within the host, as well as facilitating migration through host tissues [20,21]. Genes coding for proteins with a proven or putative ability to interact with host cells include those coding for the immunomodulatory molecules IPSE/alpha-1 and omega-1, micro-exon gene proteins (MEGs), venom allergen-like proteins (VALs), but also as shown in our recent work, proteolytic enzymes and protease inhibitors [22].

Together, these early and later -omics studies have provided the intellectual basis for how we understand the biology of the *S*. *mansoni* egg stage today. However, all canonical -omics studies analyzed liver-derived eggs despite cardinal differences in the cellular composition of periovular granuloma in the liver, colon, and ileum [10–12,20,22–24]. Although the studies of the “losers” are essential for understanding the pathogenesis of schistosomiasis, they do not provide insight into the biology of successful intestinal eggs—the "winners". Moreover, considering the recent study showing that *in vitro* laid eggs exhibit a different transcriptomic profile from those isolated from the liver [21], a fundamental question arises, namely if and possibly how the egg is affected by the surrounding tissues.

In this study, we sought to fill this knowledge gap with a comprehensive approach based on a robust comparative analysis of gene expression in mature and immature eggs isolated from both the mouse intestine and the liver. Together with these analyses, we conducted experiments to examine differences in the viability and immunogenicity of these eggs. This multifaceted approach allowed us to uncover previously unknown effects of host surrounding tissues on the development and interactions of *S*. *mansoni* eggs in the definitive host. These findings carry substantial implications for basic and clinical studies focused on the egg stage of this parasite.

## 2 Results

### 2.1 The tissue origin of *S*. *mansoni* eggs significantly affects the miracidia viability and egg gene expression

The objective of this study was to unravel potential differences in egg morphology, gene expression, viability of hatched miracidia, and the host’s immune response to soluble egg antigen (SEA) between eggs isolated from liver and intestinal tissues. Currently, this knowledge is either entirely absent or limited to liver-derived eggs, and understanding these differences can reshape our view of the parasite’s life cycle.

**Fig 1:**
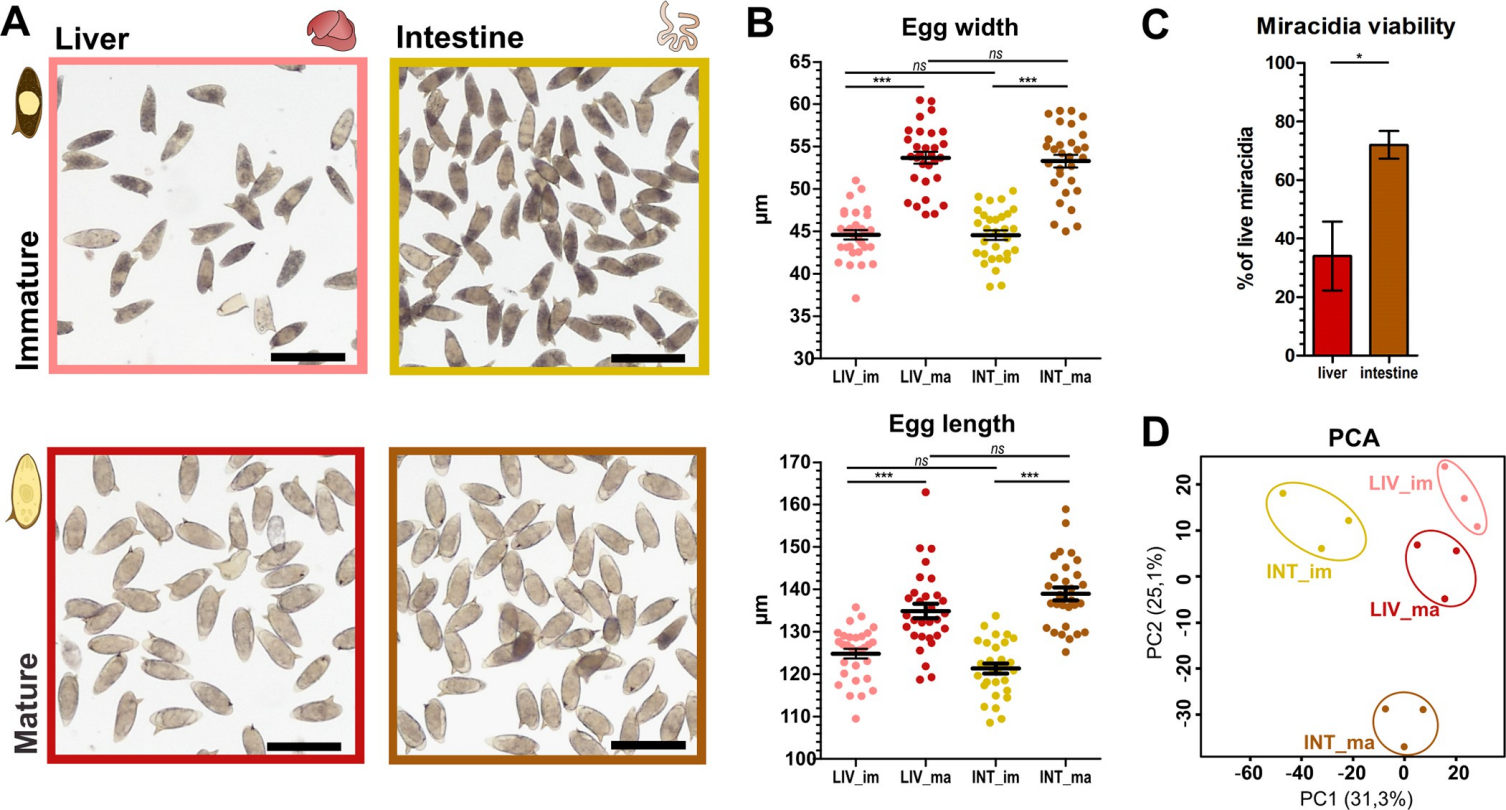
Analysis of immature and mature *S*. *mansoni* eggs isolated from mouse livers and intestines with focus on morphology, miracidia viability and differential gene expression. **(A) Microscopy visualization:** Images depict the morphology of eggs following isolation and Percoll separation, highlighting the visual differences between immature and mature stages. Scale bar = 200 μm. **(B) Size measurements:** An analysis of the length and width of *S*. *mansoni* eggs (n = 30) after separation by Percoll. The results were statistically evaluated using a Two-way ANOVA in GraphPad Prism; error bars = SD; ***p<0.001; non-significant = ’*ns*’. **(C) Viability of miracidia originating from liver and intestinal eggs:** Percentage of viable miracidia 4 hours after hatching. Bar graph shows a mean of 3 biological replicates represented by miracidia originating from three different mice. Number of miracidia observed in each liver/intestine replicate was 16–27. Data were evaluated by t-test in GraphPad Prism; error bars = SD; *p<0.05. **(D) Principal Component Analysis (PCA):** The PCA plot showcases the clustering of eggs based on organ origin and maturity level, reflecting the underlying variations in gene expression profiles analyzed through RNA-Seq. INT_im: intestinal immature eggs; INT_ma: intestinal mature eggs; LIV_im: liver immature eggs; LIV_ma: liver mature eggs.

After isolation, we separated the eggs into immature and mature groups using a Percoll gradient. Successful separation of the eggs by their maturity was confirmed by microscopic observation of the morphology of each group of eggs and supported by significant differences in measured dimensions (Fig 1A and 1B). The dimensions of eggs from the intestine and liver did not vary within the two groups. The morphology of eggs designated as *immature* in this study corresponds to stages 1–6 as categorized in the staging system proposed by Jurberg et al., (2009) [4]. In brief, freshly laid eggs at stage 1 contain an embryo and vitelline cells; after mitotic divisions and embryonic cleavages, eggs at stage 6 contain an embryo with developing glands, neural mass, epidermal epithelium, germ cells, muscle precursors and inner and outer envelopes. The other group of *mature* eggs in our study corresponds to stages 7 and 8 of development as described by Jurberg et al., (2009) [4]. These stages are distinguished by the presence of a nearly to fully developed miracidium with differentiated musculature, flame cells, and ciliated epidermal plates.

Mature eggs from the liver and intestine were stimulated to hatch, and the viability of the hatched miracidia was evaluated. Three to four hours after hatching, we observed that an average of 72% (SD ±7) of the miracidia hatched from intestinal eggs were still viable and actively moving. In contrast, only 37% (SD ±17) of the miracidia from liver eggs survived (Fig 1C). These findings indicate a significant difference in viability between miracidia hatched from intestinal eggs compared to those from liver eggs.

Next, we examined the overall gene expression profiles of immature and mature eggs from both the liver and the intestine. The RNA was extracted from the isolated eggs and subjected to Illumina sequencing for the transcriptome analysis. Raw reads are deposited and available under accession number PRJNA1036419 in NCBI GeneBank database. On average, each sample produced approximately 10.1 million raw reads (SD = 2.0 million), with an average mapping rate of 79.9% (SD = 11.3%) to the reference genome (S1 Table). With the data obtained, we conducted a differential expression analysis by comparing each egg sample group with one another. Interestingly, the analysis clearly shows that gene expression in *S*. *mansoni* eggs is critically dependent not only on their developmental stage but also on their tissue localization. Notably, the PCA cluster for mature intestinal eggs exhibited the most pronounced differences in gene expression compared to the other groups, suggesting that tissue-specific localization plays an essential role in the biology of these eggs (Fig 1D).

In summary, as expected, immature and mature eggs differ significantly in size and transcriptomic profile. At the same time, however, it is clear that although eggs from the liver and intestine appear visually identical at first glance, there is a significant difference in their gene expression and then even in the viability of the hatched miracidia.

### 2.2 Gene expression changes during egg development are reflected in pathways associated with mitochondria, ribosomes, organelles, and cilia assembly

In order to better understand the gene expression in eggs based on their tissue origin and stage of development, we analyzed and compared transcriptomic profiles of immature and mature eggs that were extracted directly from the intestine and liver of experimentally infected mice.

In all egg groups examined, the most highly expressed transcripts were associated with genes involved in the mitochondrial electron transport chain (namely *cox1-3*, *cob*, *atp6* and *atp8*, *nad1-6* and *nad4L*) and with translation processes in ribosomes (e.g., elongation factor 1-alpha, large ribosomal subunit protein P2, and 40S ribosomal protein S30) (see S2 Table). These findings underscore their fundamental role in egg development and survival.

**Fig 2:**
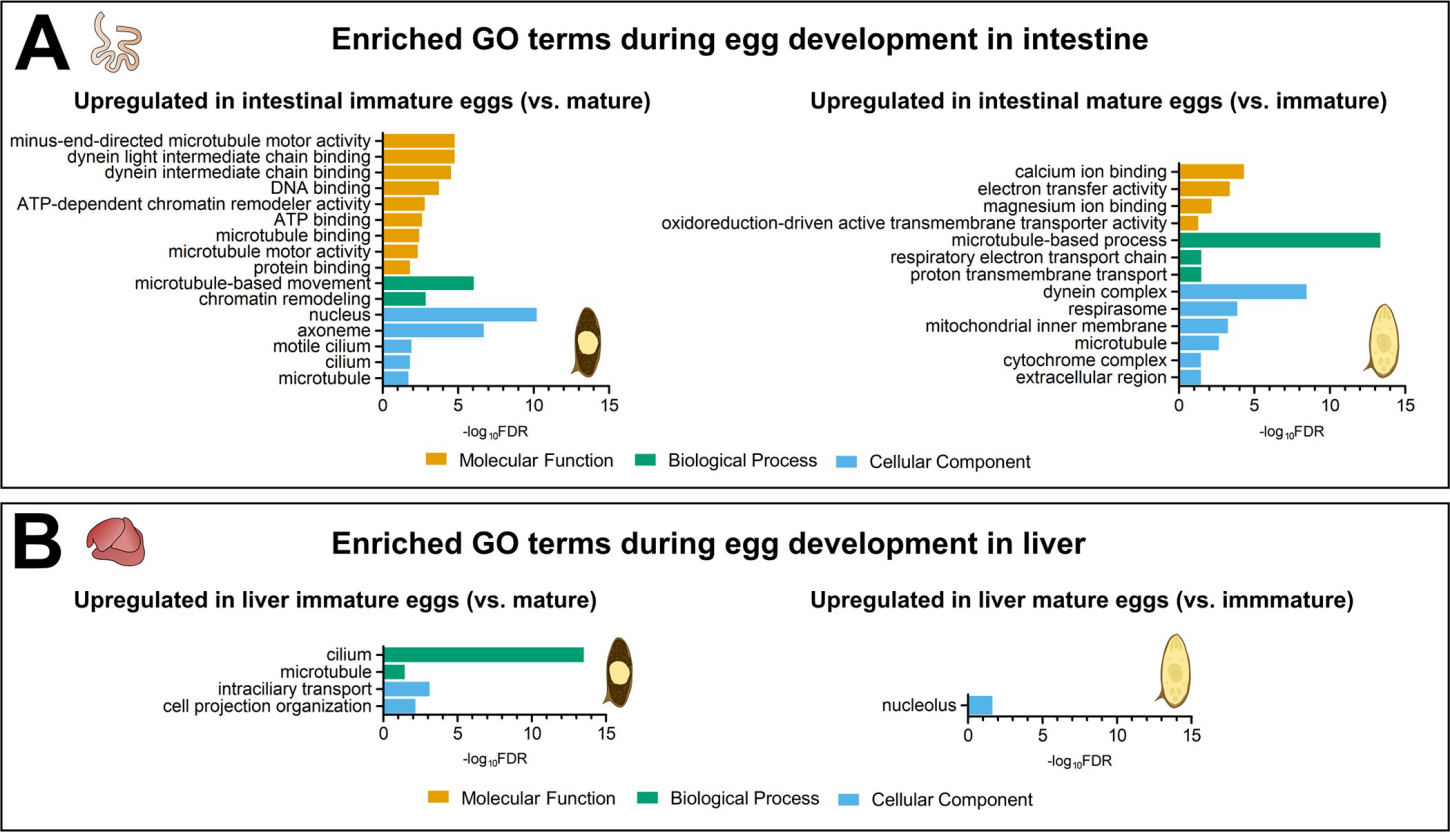
Comparative Gene Ontology (GO) enrichment analysis between immature and mature *S*. *mansoni* eggs. **(A)** Upregulated pathways of immature and mature eggs developing in the murine intestine. **(B)** Upregulated pathways of immature and mature eggs developing mouse liver. The plots are segmented into three sections for Molecular function (orange), Biological process (green), and Cellular component (blue). A maximum of ten pathways from each category are shown.

To understand the transcriptional changes at different developmental stages, we performed a differential gene expression analysis comparing immature and mature eggs. Separate comparisons were made for eggs from the intestine and liver. In intestinal eggs, we identified 1304 differentially expressed genes (DEGs) with a padj < 0.05 and a log2 fold change > 1, indicating statistically significant upregulation. Of these, 710 were upregulated in immature eggs, while 594 were upregulated in mature eggs (S3 Table). By classifying these genes according to their Gene Ontology (GO), we identified enriched functional pathways in each group. For immature intestinal eggs (compared to mature eggs), we identified 16 enriched GO terms, and the most prominent ones included "nucleus", "axoneme", "microtubule-based movement", and "chromatin remodelling". These terms are associated with growth, egg embryo development, and the onset of growth of the rich ciliature characteristic of the miracidium surface (Fig 2A and S4 Table). Mature intestinal eggs were enriched in 13 GO terms, among which were "microtubule-based process", "calcium ion binding", "dynein complex" and "respirasome", which correspond to the increasing energy demand as the miracidium begins to activate muscles and cilia (Fig 2A and S4 Table).

When we compared immature and mature liver-derived eggs, 1191 DEGs were identified: 547 upregulated in immature eggs and 644 in mature eggs. Comparative GO enrichment analysis identified less up- and downregulated GO terms than in intestinal eggs. Immature liver eggs had 4 enriched GO terms that mostly overlapped with those of immature intestinal eggs, such as "cillium", "microtubule", and "intraciliary transport" (see Fig 2B and S4 Table). The mature liver eggs exhibited only one enriched GO term: “nucleolus”. Notably, this GO term was not upregulated in mature intestinal eggs, suggesting that the specific host tissue environment influences gene expression during egg development.

To summarize, there is a significant shift in gene expression between immature and mature eggs, regardless of whether they develop in the intestine or the liver. Changes in immature eggs predominantly relate to embryogenesis and growth, whereas mature eggs exhibit diverse upregulated pathways.

### 2.3 Intestinal eggs exhibit remarkable upregulation over liver eggs in gene expression, protein synthesis, and mitochondrial activity pathways

**Fig 3:**
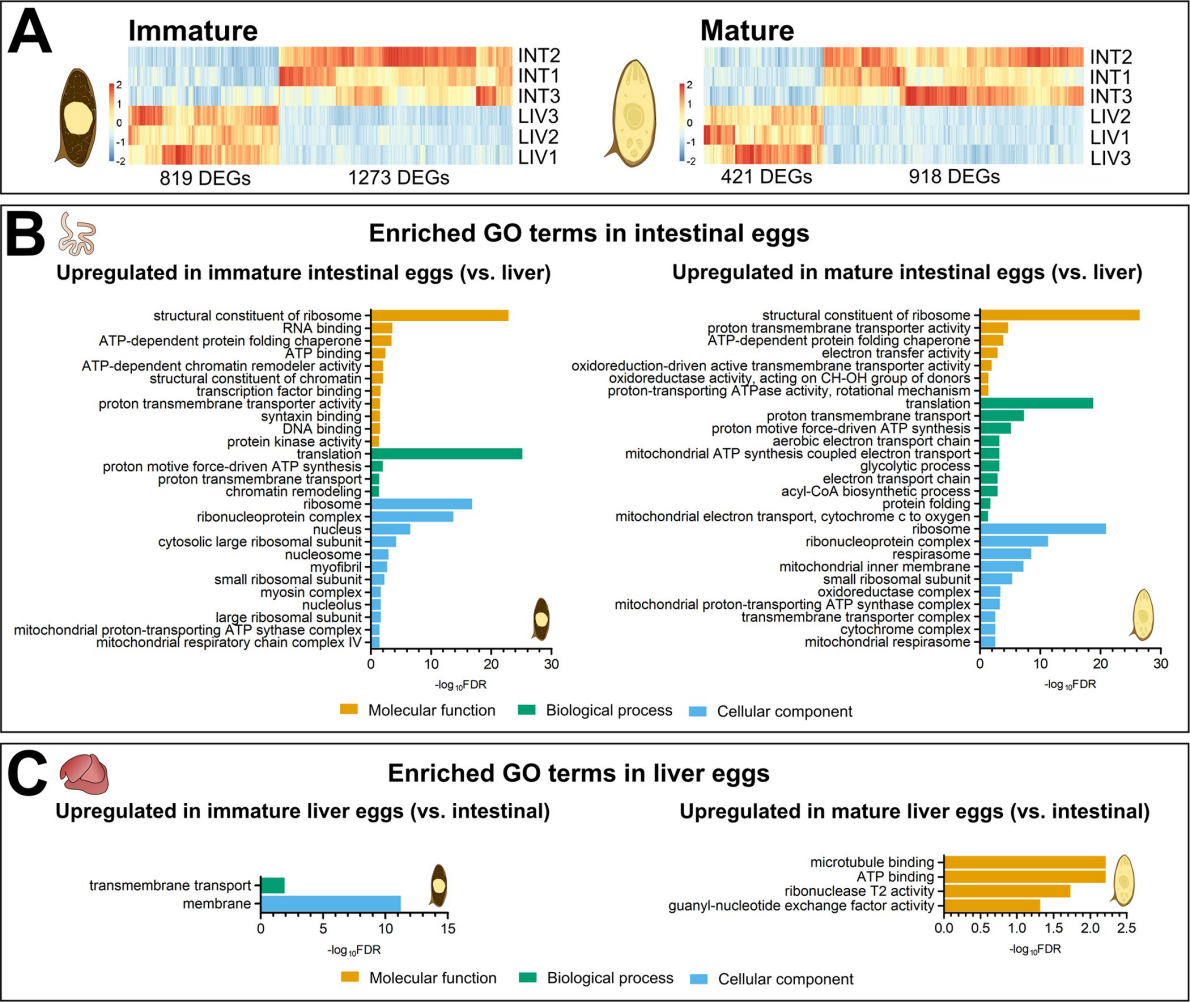
Differential expression analysis (DESeq2) and Gene Ontology (GO) enrichment analysis comparing *S*. *mansoni* eggs isolated from intestine and liver. **(A)** Heatmaps show the overview of all differentially expressed genes (DEGs) between eggs isolated from the intestine and liver, encompassing both developmental stages: immature and mature eggs. Notably, intestinal eggs have more upregulated genes in both developmental stages. INT = intestinal eggs; LIV = liver eggs. **(B, C)** Comparative GO enrichment analysis between intestinal (B) and liver (C) eggs. The plots are segmented into three sections for Molecular function (orange), Biological process (green), and Cellular component (blue). A maximum of ten pathways from each category are shown.

The primary objective of this study was to determine whether there is a difference in gene expression between eggs isolated from the intestine and liver—the two main host organ tissues where the eggs are deposited. We found that eggs of intestinal origin exhibited differential expression in approximately twice as many genes, both immature and mature, compared to liver-derived eggs (Fig 3A). This suggests that intestinal eggs have upregulated expression in a broader range of genes compared to liver eggs.

After functional categorization of DEGs using GO terms and enrichment analysis (S4 Table), we observed that intestine-derived immature eggs were enriched in 27 GO terms (in comparison to immature liver-derived eggs), with the most pronounced enrichment associated with translation and gene expression processes (Fig 3B). In contrast, immature eggs from the liver were enriched in only 2 GO terms: “membrane” and “transmembrane transport” (Fig 3C). In the case of mature eggs, those isolated from the intestine showed enrichment in 38 GO terms. While some of these terms remained related to translation as observed in immature eggs, we also identified additional terms, in particular, related to mitochondrial functions, electron transport, and cellular respiration (Fig 3B). On the other hand, mature liver eggs exhibited enrichment in 4 GO terms among which were “microtubule binding”, “ATP binding” and “ribonuclease T2 activity” (Fig 3C).

Having identified the enriched pathways, we examined the transcript levels of individual genes that were highly expressed. Notably, there was a pronounced up-regulation of genes associated with mitochondria in intestinal eggs, particularly those encoded by mitochondrial DNA (mtDNA), (S3 Table). To further validate this observation, we examined the overall proportion of transcripts in each egg group. Specifically, immature intestinal eggs contained 8.3% of their transcripts corresponding to mtDNA genes, while mature eggs had 22.7%. In contrast, the proportions for immature and mature liver eggs were only 2.0% and 1.4%, respectively. The genes encoded in mtDNA are primarily associated with aerobic metabolism for ATP production. The marked difference in their expression between liver- and intestine-derived eggs suggests that tissue localization of eggs strongly influences egg energy metabolism and that intestinal eggs may have enhanced aerobic metabolism. Looking at the enzymes responsible for anaerobic ATP production by glycolysis, we observed high transcription of glycolytic enzymes (e.g., glyceraldehyde-3-phosphate dehydrogenase, hexokinase, phosphoglycerate mutase) in eggs. These did not show an overall change in expression levels during egg development but were also upregulated in intestinal eggs. Several enzymes of glycolysis (defined by identification of GO term: 0006096; glycolytic processes) showed significantly increased expression in intestinal eggs according to DESeq2 analysis, specifically 3/12 in immature and 7/12 in mature eggs (S5 Table).

Overall, our findings show that intestinal eggs exhibit prominent upregulation of genes associated with translation and energy metabolism, whereas liver eggs exhibit upregulation membrane-associated activity, and interestingly ribonuclease T2 activity.

### 2.4 Genes potentially involved in egg-specific interaction with the host are regulated based on their tissue localization

To make the most of the obtained data, we also narrowed our focus on the expression of genes whose protein products are potentially important for egg-host interactions but have been rather neglected in experimental parasitology. Those include Venom allegen-like proteins (VALs) that seem to be strongly linked to parasitism, especially in helminths, where the encoding genes are significantly amplified in their genomes [25], and micro-exon genes (MEGs), that are in turn unique for their gene structure and have only been identified in parasitic platyhelminths [26,27]. Moreover, representatives of both of these groups were identified in the ESPs of *S*. *mansoni* eggs and are therefore considered to be in direct contact with the host’s tissues [10,11].

**Fig 4:**
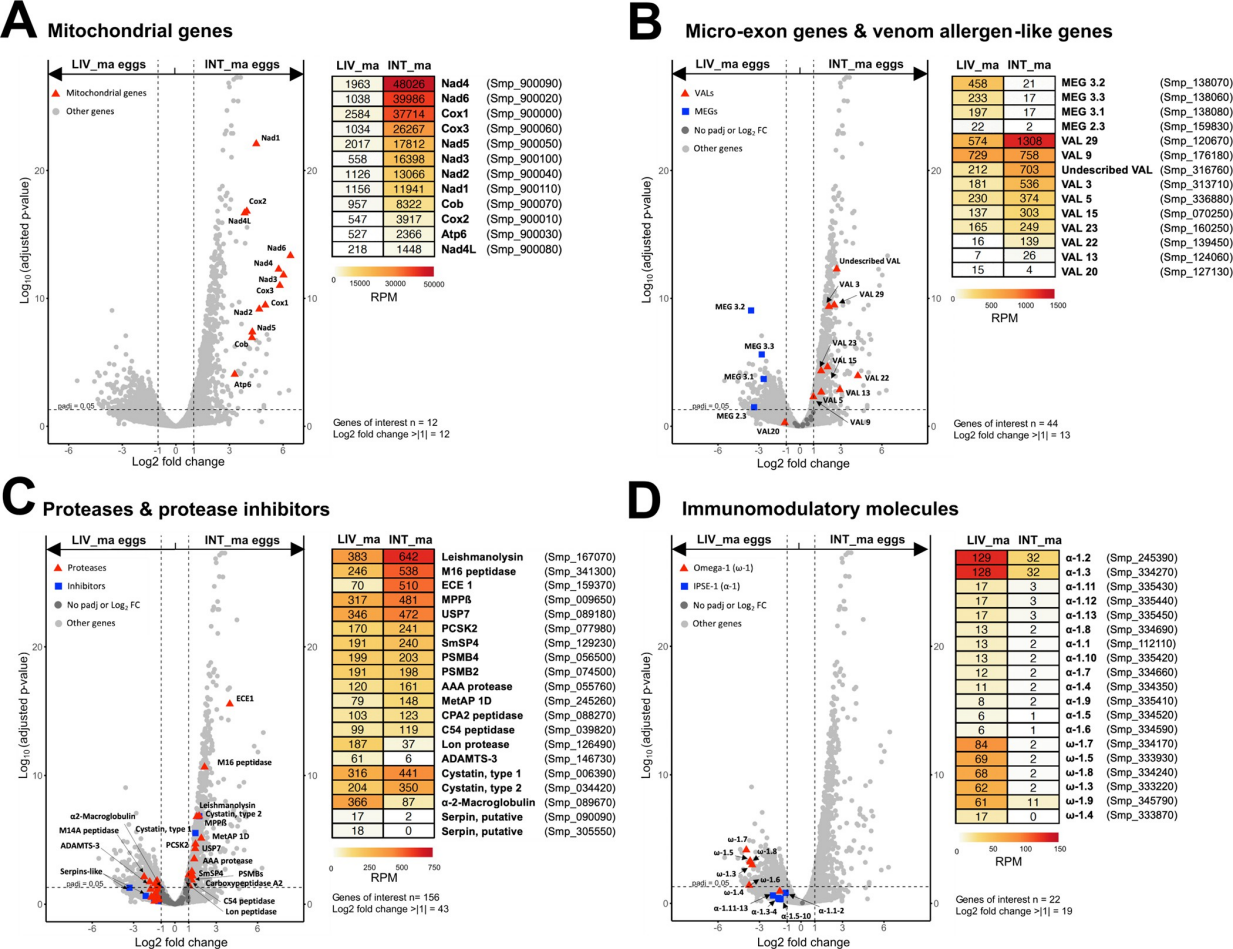
Comparative gene expression analysis, investigating mitochondrial genes (A) and genes encoding molecules with potential roles in egg-host interaction (B, C, D) within mature *S*. *mansoni* eggs obtained from the intestinal and liver tissues of experimentally infected mice. Volcano plots show genes that were statistically significantly differentially expressed (DESeq2 analysis; padj < 0.05; log2 fold change > 1) This encompasses (**A**) genes encoded in mitochondrial genome, (**B**) micro-exon genes (MEGs) and venom allergen-like proteins (VALs), (**C**) immunomodulatory molecules IPSE/alpha-1 and omega-1 and (**D**) proteases and inhibitors in *S*. *mansoni* eggs. The corresponding heat maps show a mean of gene expression levels normalized to Reads Per Million (RPM), serving to highlight the proportional contributions of each subgroup to the overall gene expression profile in a given sample.

We observed that both VALs and MEGs have very distinct expression patterns between mature eggs derived from liver and intestinal tissues (Fig 4B). While differentially expressed MEGs were upregulated in mature liver eggs, VALs were highly dominant in mature intestinal eggs. This did not apply only to MEG-9 and VAL20, but both genes had negligible expression levels in all samples of ≤ 15 Reads per million (RPM). Of the 24 unique MEGs selected (S5 Table), six genes exibited statistically significant differential gene expression between mature eggs from different tissues ranging from 1.4 to 3.6 log2 fold change in favor of liver eggs. Three of these genes belonged to the MEG-3 family and three to the MEG-2 family. The expression of MEGs in eggs was relatively high, with MEG-6 showing the highest expression of 614 RPM in mature liver eggs. In the version of the v9 genome that we worked with, we identified 35 genes that encode the complete SCP/TAPS domain (Sperm-coating protein/ Tpx-1/Ag5/PR-1/Sc7) (S5 Table) and can thus be considered VAL proteins. Of these 35 genes, nine were significantly differentially expressed with log2 fold change ranging between 1.5 and 4.2 in favor of the intestine-derived eggs. From 12 highly expressed VALs (RPM > 50) in either group, 8 VALs were statistically differentially expressed, and all of these were upregulated in the intestinal-derived eggs. The relatively most expressed of among all genes coding VALs and MEGs was VAL29, exceeding 1300 RPM in intestinal eggs, while the most statistical difference in gene expression between tissues was for a previously undescribed VAL (Smp_316760). Interestingly, while MEG proteins exhibit a liver-egg expression preference from the early stages of egg development, VAL proteins only display intestinal-egg-specific expression after reaching maturity (S1 Fig).

We also examined the expression patterns of proteases and protease inhibitors as molecules frequently involved in host-parasite interactions [28,29]. Genes included in differential expression analysis were selected based on our previous research comparing gene expression profiles of proteases and their inhibitors in *S*. *mansoni* and *Fasciola hepatica* eggs [22]. Of the 129 proteases analyzed, we observed 24 significantly differentially expressed, out of which 16 and 8 were upregulated in isolated eggs from intestine and liver, respectively (Fig 4C). The most expressed protease in both samples was leishmanolysin-like peptidase, while the biggest statistical difference in expression was observed in endothelin-converting enzyme 1 (ECE1), the third most expressed protease in the intestinal eggs. Of the 21 protease inhibitors considered, only two were significantly upregulated, namely, cystatins type-1 and type-2, and both of these genes were upregulated in eggs of intestinal origin. Cystatin type-1 was also the most expressed protease inhibitor in intestinal eggs. Genes encoding two potential serpin inhibitors and alpha-2-macroglobulin were very close to reaching the threshold for statistical significance (with adjusted p-values of 0.05, 0.05, and 0.07, respectively). Despite low gene expression of both serpins in eggs from both tissues, alpha-2-macroglobulin was the inhibitor with the highest expression in liver eggs, reaching 366 RPM.

Taken together, eggs from the liver and intestine exhibit major differences in expression patterns of VALs and MEGs. MEGs appear to be liver-specific, while VALs are dominant in the intestinal eggs. While proteases and inhibitors did not show such distinct patterns, significantly upregulated genes tend to be more highly expressed in intestinal eggs.

### 2.5 Egg immunomodulatory molecules are preferably expressed in liver as supported by direct observation of IPSE/alpha-1

We also observed a remarkable tissue-dependent difference in the expression of two important immunomodulatory molecules IPSE/alpha-1 and omega-1 (Fig 4D). These proteins play pivotal roles not only in orchestrating a shift toward a Th2 immune response within the host but also in mediating the subsequent pathologies associated with the dissemination of parasite eggs to non-specific tissues by inducing granuloma formation [16,30,31]. Notably, IPSE/alpha-1 also stands out as the sole protein whose presence and secretion have been confirmed through immunolocalization within liver granulomas [13,30,32]. In v9 *S*. *mansoni* genome, IPSE/alpha-1 is encoded by a set of 13 paralogous genes, while omega-1 is encoded by 9 distinct genes (S6 Table). Intriguingly, our DESeq2 analysis revealed that five of the omega-1-encoding genes (omega-1.3, omega-1.5, omega-1.6, omega-1.7 and omega-1.8) demonstrated statistically significant upregulated expression in mature liver eggs (padj < 0.05). Although all 13 of the IPSE/alpha-1 encoding genes exhibited notable upregulation (log2 fold change > 1) in favor of mature liver-derived eggs, the differential expression of these genes fell just below the threshold of statistical significance (padj > 0.05). Interestingly, a similar trend was also observed in immature eggs, where six IPSE/alpha-1 genes (IPSE-1, IPSE-8, IPSE-10, IPSE-11, IPSE-12, IPSE-13) and one omega-1 gene (omega-1.4) were significantly differentially expressed in favor of the eggs isolated from the liver. It is also worth mentioning, that although just below the threshold for statistical significance, here too all IPSE/alpha-1 and omega-1-coding genes had increased expression in the liver eggs (S1 Fig).

**Fig 5:**
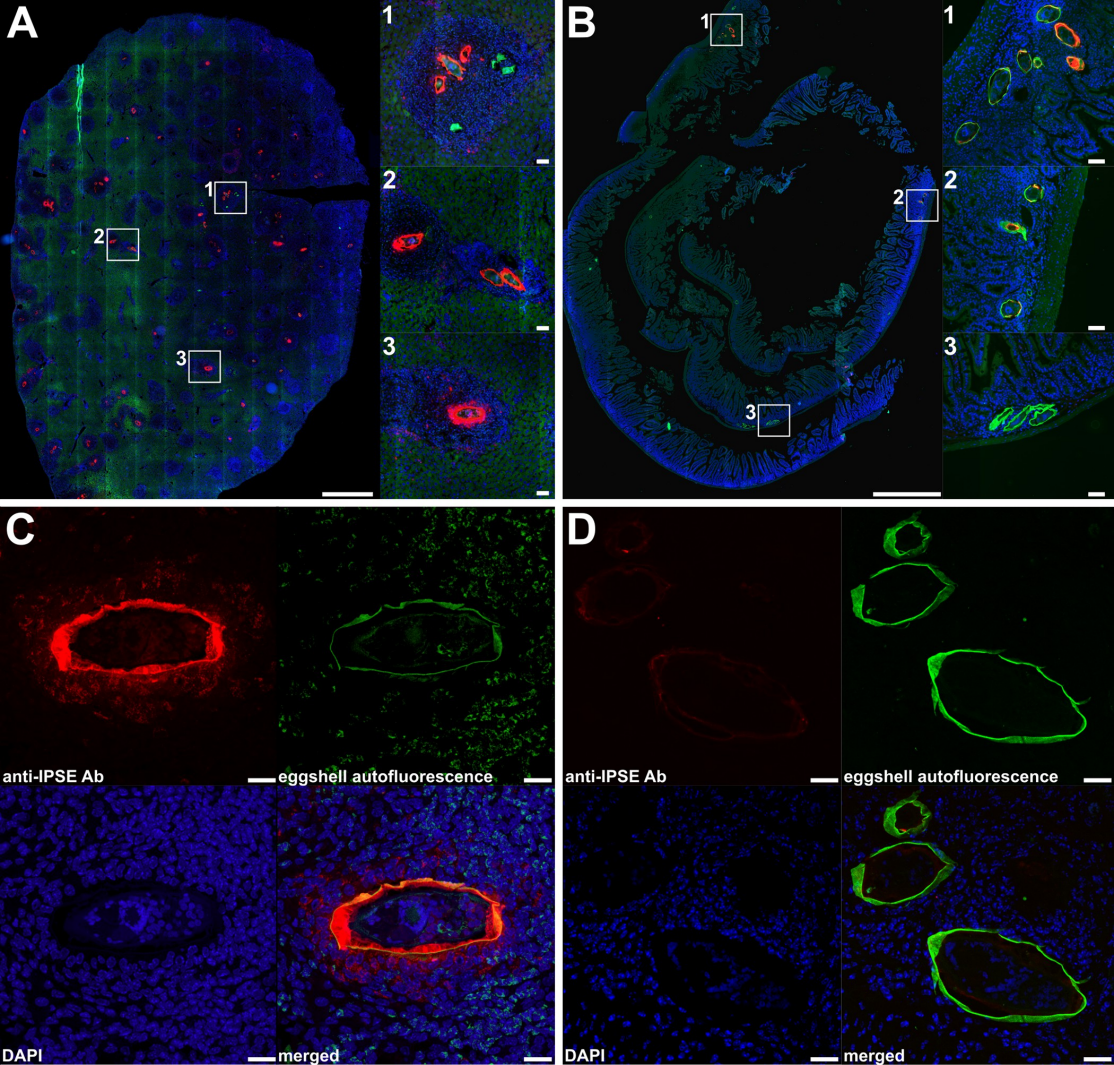
Immunolocalization of IPSE/alpha-1 in mouse liver (A, C) and intestine sections (B, D) infected with *S*. *mansoni*. **(A, B)** Overview axioscan images display entire sections of infected tissue, with three highlighted zoomed areas. **(C, D)** Confocal close-ups illustrate mature eggs with developed miracidia. Labels are represented by red for anti-IPSE/alpha-1 antibody-specific sites, green for eggshell autofluorescence, and blue for DAPI-labeled cell nuclei. The red anti-IPSE/alpha-1 signal is particularly intense in the subshell area and around liver eggs, while it is weaker or absent in the intestinal eggs. Scale bars: whole-organ section = 1000 μm, zoomed square areas = 50 μm, and confocal close-ups = 20 μm. For negative control images see S2 Fig.

To further support our transcriptomic data, we conducted immunolocalization experiments to examine the presence of IPSE/alpha-1 in eggs embedded within both of the tissues under investigation. Although whole sections of both organs were analysed (Fig 5A and 5B), we also paid special attention to eggs containing visibly differentiated miracidia, where genes encoding IPSE/alpha-1 showed preferential upregulation in the liver but fell just below statistical significance. This was done to further strengthen our data interpretation and make our conclusions comparable with previously published results [13,30,32]. These experiments indeed showed that IPSE/alpha-1 is produced and secreted from eggs in the liver and internalized by surrounding granuloma host cells (Fig 5A and 5C), as has been shown in the past [13,30]. The localization of IPSE/alpha-1 production corresponded to the subshell envelope, where all secreted protein production is suspected to occur [3]. The localization of IPSE/alpha-1 also overlapped with the autofluorescence produced by the eggshell, indicating its passage into surrounding tissues where the signal fades with distance from the egg (Fig 5C). No significant signal was observed in the miracidium (Fig 5C). On the contrary, the signal of IPSE/alpha-1 in and around intestinal eggs was detected only sporadically and at lower intensity (Fig 5B and 5D). The overall pattern was consistent across the whole tissue sections, as shown in the axioscan photos (Fig 5A and 5B).

Thus, these results agree with our bioinformatic data, and the combined evidence suggests that key host immunomodulation factors omega-1 and IPSE/alpha-1 are differentially expressed in intestinal and liver eggs showing substantial upregulation in the latter.

### 2.6 Intestinal and liver SEA elicit different host immune reactions

**Fig 6:**
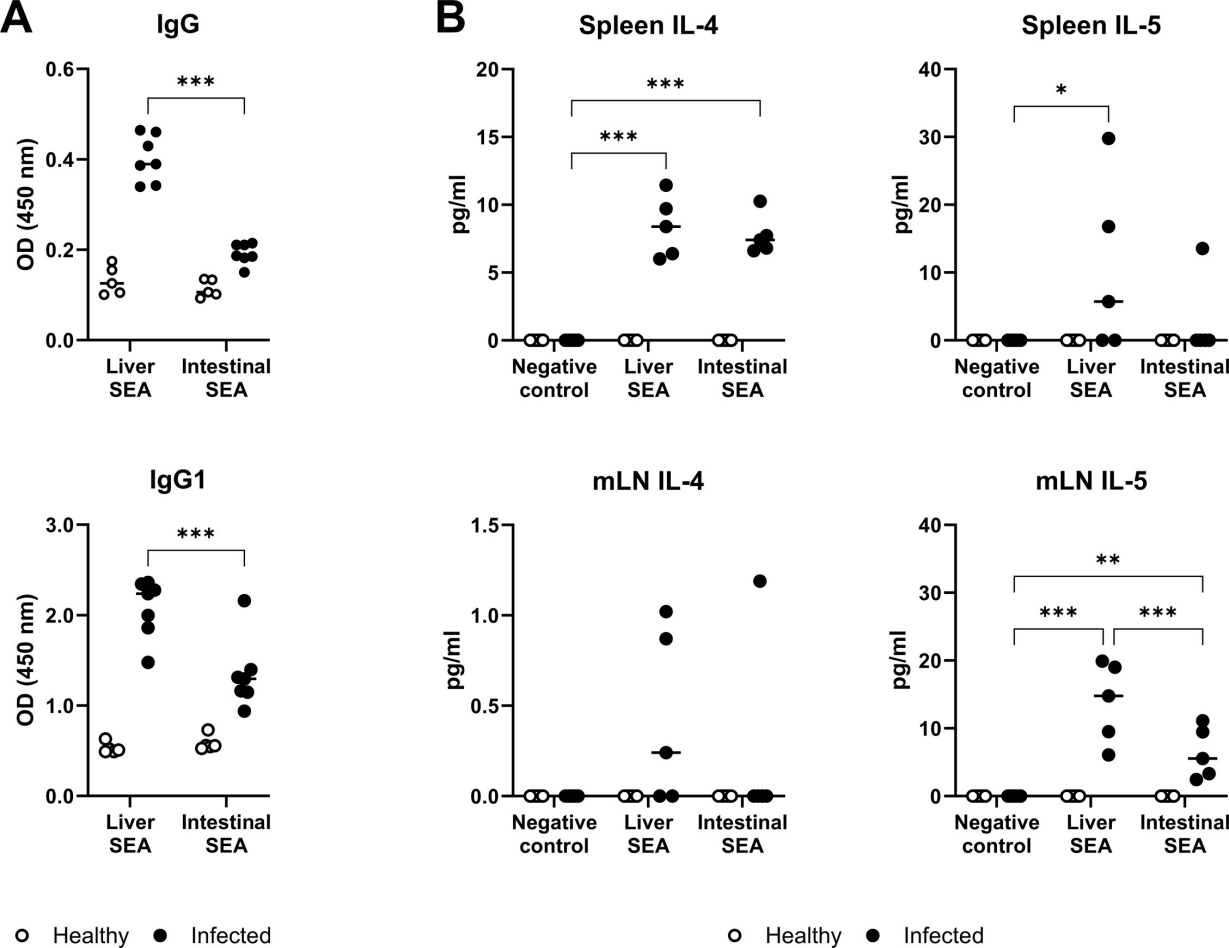
Soluble egg antigens (SEA) derived from liver and intestinal eggs differentially affect the host immune response. Mice were infected with 150 cercariae of S. mansoni. Serum, spleen, and mesenteric lymph nodes (mLNs) were collected at 7 weeks post infection. Uninfected (healthy) age-matched mice were used as controls. (A) Reactivity of serum antibodies (IgG and IgG1) with liver and intestinal SEA. (B) Cytokine production by splenocytes or mLN cells treated by liver or intestinal SEA (20 μg/ml) for 72 hours. Values obtained from individual mice are shown (n = 5–7). Data were evaluated by 2-way ANOVA followed by Holm-Šídák’s multiple comparisons test; *p<0.05, **p<0.01, ***p<0.001.

Our transcriptomic and immunolocalization data demonstrated distinct expression of key immunomodulatory molecules (IPSE/alpha-1, omega-1) in intestinal and liver eggs. Hence, we assessed whether SEA from intestinal or liver eggs have different capacities to elicit the host immune response. First, we noticed lower recognition of intestinal SEA by antibodies in the sera of infected mice. Specifically, levels of complete intestinal SEA-specific IgG and Th2-associated IgG1 were significantly reduced (Fig 6A). This suggests dissimilar antigen composition of intestinal and liver SEAs, resulting in distinct antigenicity. Next, we tested the effect of SEA on cytokine production by lymphocytes as they orchestrate the host immune reaction and tissue pathology. To test both systemic and local effects, we isolated cells from spleens and mesenteric lymph nodes (mLNs), respectively. While splenocytes and mLN cells produced similar amounts of IL-4 after treatment by intestinal and liver SEA, the production of IL-5 was tendentially lowered in splenocyte cultures (p = 0.09) and significantly reduced in mLN cultures treated by intestinal SEA (Fig 6B). This indicates unequal effects of liver and intestinal SEAs on inducing the antigen-specific T cell response.

## 3 Discussion

*S*. *mansoni* eggs have long been the subject of intense investigation for their central role in the pathology associated with chronic schistosomiasis. However, despite decades of these research efforts, the studies have mostly focused on so-called “loser” eggs trapped and dying in the liver, while “winner” eggs passing through intestinal tissue, essential for the life cycle to continue, have historically been neglected. Although fundamental differences in size, cellularity, and extracellular matrix content were reported for liver and intestinal periovular granulomas 40 years ago [23,33], a detailed insight into the differences in egg biology concerning their tissue origin have never been pursued further.

In the present study, we filled this gap with a robust comparative transcriptomic analysis showing pronounced differences between intestinal and liver-entrapped eggs. The primary findings of variability in gene expression between eggs were supported by experiments confirming that gene expression data are consistent with protein production, as shown by exemplary localization of IPSE/alpha-1. Moreover, we revealed that “winner” and “loser” eggs elicit distinct immune responses *in vitro*, and hatched miracidia have different viability.

The first aim of this study was to investigate gene expression changes in eggs by comparing immature and mature eggs from both organs during their ontogenetic development. While this has been extensively studied and well described microscopically [4], investigations of gene expression changes during this phase have mainly focused on *in vitro* cultured eggs, which lacked host stimuli [21] or eggs derived only from liver tissue regardless of developmental stage [20]. Our results demonstrate that a high proportion of genes upregulated in immature eggs are related to embryonic development and miracidial cilia differentiation. These patterns were consistent in both intestine- and liver-derived immature eggs and align well with findings from *in vitro* laid eggs previously studied by Lu et al. (2021) [21]. This suggests that the surrounding tissue has a minimal influence on the early miracidia development. This is also supported by the fact that the eggs from both tissues are morphologically alike and give rise to viable miracidia. However, miracidia hatched from liver eggs had a shorter lifespan than those hatched from the intestinal eggs, suggesting that the massive inflammatory reaction [34] or other liver tissue factors, may have a negative influence on larval fitness. However, this effect may also be caused by the fact that while intestinal “winner” eggs are likely to leave the host soon after maturation, mature liver eggs likely remain viable in the host tissues for an extended period. This may consequently lead to reduced vitality of fully formed larvae. Findings about higher viability of the miracidia hatched from intestinal eggs may also have implications for the routine maintenance of the parasite’s life cycle. This task is often challenging and the liver eggs are typically used for this purpose [35,36].

Mature eggs generally showed differentially enriched pathways when comparing early to later developmental stages. In mature liver eggs, specific GO terms related to calcium and metal ion transport were more abundant than in immature eggs. On the other hand, mature eggs from the intestine were enriched in pathways related to mitochondrial processes and energy production compared to immature eggs. This suggests that surrounding tissues may have a greater influence on gene expression over time, although the precise interpretation of the enriched pathways requires further investigation.

When comparing eggs based on their tissue of origin, we discovered significant variations in their gene expression. One of the most distinctive difference is the marked upregulation of genes related to aerobic energy metabolism of mitochondria (especially those coded by mtDNA) in mature intestinal eggs vs. mature liver eggs. Although we currently lack a definitive explanation for this novel finding, we believe our data provide a strong basis for future research regarding egg energy metabolism. Another interesting difference was that liver eggs displayed notably higher gene expression of omega-1 and IPSE/alpha-1, well-established immunomodulatory molecules that promote the shift from Th1 to Th2 immune response [14] and release of IL-4 and IL-13 from basophils, respectively [32]. Indeed, the remarkable upregulation of the T2 ribonuclease omega-1 [16] was reflected in the GO enrichment of RNA nuclease-related pathways in the liver eggs. We validated our transcriptomic data by immunolocalization of IPSE/alpha-1 protein. It was prevalent in the subshell envelope of liver eggs and also in the adjacent liver parenchyma, thus corroborating with the previous finding [13], but less often observed in the intestinal eggs or tissues. As both omega-1 and IPSE/alpha-1 are potent immunomodulators facilitating granuloma formation [15,37], our data on their differential expression could explain the dissimilar features of liver and intestinal periovular granulomas [6,23,33]. These differences have been previously attributed to the unequal capacity of the tissues to mount immune reactions and/or to parasite-related factors [21,24]. Here, we present comprehensive data supporting the view that the decreased production of these immunomodulators by intestinal eggs is responsible for the alleviated granuloma formation in the intestine. Beyond the much lower expression of immunomodulators omega-1 and IPSE/alpha-1 by intestinal eggs, our experimental data also show that intestinal SEA elicited lower IL-5 production by mLN cells.

Considering the role of IL-5 in the development and recruitment of eosinophils [38,39], we suggest that intestinal eggs have a decreased capacity to initiate eosinophil recruitment into the granulomas as already reported in the past [23]. Our observation of mitigated antibody response against intestinal SEAs underpins the dissimilarity between liver and intestinal eggs. We suggest that downregulated expression of omega-1 and IPSE/alpha-1 antigens strongly recognized by the host antibodies [17] could be responsible for the discrepancy between liver and intestinal SEA antigenicity. Admittedly, the observed differences in the immunogenicity/antigenicity of intestinal and liver SEA used in our experiments could be partially ascribed to the unequal ratio of mature and immature eggs in liver and intestinal tissues (with the liver containing more mature eggs permanently trapped in the tissue). However, our data show no significant differences in mature/immature egg ratios or egg counts (S3 Fig). And even if so, it would represent the actual tissue egg burden, which is what is exposed to the host’s immune response.

Altogether, our transcriptomic and experimental data demonstrate that mature liver and intestinal eggs of *S*. *mansoni* differ in factors influencing the host immune system. We speculate on the reason or purpose of this phenomenon. As the canonical immunomodulators omega-1 and IPSE/alpha-1 are expressed chiefly in “loser” liver eggs, we speculate that the expression is boosted by yet to be defined liver-specific tissue factors leading to the formation of granulomas preventing excessive tissue damage [8]. This is accompanied by Th2-driven alternative macrophage activation that helps regulate inflammation and mitigate liver pathogenesis [40] caused by trapped eggs. On the contrary, the “winner” eggs have a different strategy: to leave the host, causing no or minor damage to the intestinal wall and restricting inflammation induced by invading intestinal bacteria. While the particular passage mechanisms remain mostly undiscovered [6] it is clear that the intestinal granulomas are smaller and consist primarily of macrophages accompanied by only a few lymphocytes and eosinophils, and sparse collagen fibers [23,33]. From the evolutionary perspective, we propose that the *S*. *mansoni* lineages capable of the tissue-specific expression of omega-1 and IPSE/alpha-1 were favored as they limited the liver injury and promoted survival of the host while maintaining the exit strategies in the intestine. It is tempting to speculate whether the observed phenomenon might be analogous to that recognized in *Litomosoides* nematodes, where life cycle non-continuing "losers” indirectly assist the "winners" by modulating host immune response in their favor [41].

Tissue-specific expression in eggs was also prominent in other gene groups potentially implicated in egg-host interactions, specifically, VALs and MEGs. VALs have garnered significant attention in the study of parasitic helminths. Although their exact functions remain elusive, there is evidence suggesting that they may be involved in tissue migration [42,43], immunomodulation [44], and even structural functions [45]. However, recent research has contested conventional perspectives by characterizing egg-specific VALs as "larval transformation proteins". This conclusion was based on their heightened expression during *in vitro S*. *mansoni* egg development and their release during the miracidium-to-sporocyst transformation, supposedly emphasizing their developmental role [21,46]. Data presented in this study are consistent with available literature [21,47] that VALs are proportionally highly abundantly expressed genes in *S*. *mansoni* eggs. Distinct organ-specific patterns, such as VAL2 and VAL27 upregulation in immature liver eggs and VAL22 in immature intestinal eggs, become even more pronounced in mature eggs where differentially expressed VALs are almost exclusive for intestinal eggs. Given the unique tissue- and developmentally-specific expression of some of these genes, which begins as soon as the egg reaches the organs, the data presented here challenge the notion that the egg-specific VALs are solely miracidial proteins or "larval transformation proteins". On the contrary, this expression dependence on tissue localization rather indicates that different VAL proteins have different functions, probably associated with host-parasite interactions. The strong upregulation of certain intestinal egg-specific VALs might also support the previously ascribed role of these proteins in host tissue remodeling and subsequent translocation of the egg to the intestinal lumen [43].

MEGs are also very highly expressed genes in *S*. *mansoni* eggs. Their unique structure, consisting of short exons (≤ 36 bases) in multiples of three bases, is found in several Platyhelminthes species [26,27]. This structure allows for the expression of numerous gene variants, leading to the hypothesis that MEGs may contribute to immunomodulation or immune evasion, similar to variant surface glycoproteins in trypanosomes or var genes in plasmodia [27]. This hypothesis was also supported experimentally, wherefrom the esophagus of schistosomula and adults was shown to interact with human calgranulin S100A9, a protein with a role in promoting and regulating inflammatory processes [48]. In addition to confirming previous findings from liver-derived eggs, where members of MEG-2 and MEG-3 families were identified as dominant [11,27], our data also show MEG-6 expression in both immature and mature eggs. However, MEGs expression is minimal in intestinal eggs. With dominant gene expression in the liver eggs, together with their transcription localized in the subshell envelope [49] and their presence in liver egg ESPs [11], MEGs conspicuously resemble patterns of IPSE/alpha-1 and omega-1. Hence, one could make a compelling case that MEGs might serve analogous functions to established immunomodulators in the liver, thereby enhancing the parasite’s ability to adapt to the immunologically challenging environment, but this hypothesis requires further investigation.

In our prior publication, we investigated the role of proteases and inhibitors in *S*. *mansoni* liver eggs as potential contributors to egg-host interactions [22]. Given our focus on liver eggs at the time, our current research compels us to reevaluate these findings. While we did not observe a clear overall organ-based expression pattern, individual proteases and protease inhibitors exhibit intriguing organ-specific trends. For instance, the endothelin-converting enzyme (ECE1), previously noted as upregulated in immature versus mature liver eggs, maintains this trend in our analysis. However, in organ-specific comparisons, we found ECE1 to be the most significantly differentially expressed protease, favoring mature intestinal eggs. The decline of its expression in eggs trapped in liver tissue, coupled with its increase during intestinal egg maturation, supports our previously proposed hypothesis that ECE1 aids egg translocation through modulation of vasoconstriction [22]. Another example is leishmanolysin-like peptidases, which had previously been identified as highly expressed in liver mature eggs. After adding intestinal eggs, we observed that some genes encoding these peptidases were significantly upregulated in the intestinal compared to liver eggs. Assuming that mature intestinal eggs move towards the intestinal lumen while mature hepatic eggs remain trapped, our data support the previously ascribed role of these peptidases in migration through host tissues [50–52]. Considering the milder inflammation around intestinal eggs, this finding also supports the previously ascribed role of leishmanolysin-like peptidases in avoiding complement-mediated immune responses [53]. Altogether, our data not only corroborate our previously published findings [22] but also underscore the value of incorporating intestinal eggs into the equation, providing fresh insights into degradome-related host-parasite interactions.

The data we presented highlight the importance of considering often overlooked elements when drawing general conclusions. As stated in the introduction, the majority of studies on *S*. *mansoni* eggs have predominantly focused on liver eggs. While these findings are indeed valuable for forming a comprehensive understanding, many published studies have extended the results from liver eggs to other host organs, despite well-documented differences in tissue granuloma responses [23,33]. This drawback is not only limited to the previous large -omics studies [10–12]. For example, previous research associated SEA from liver eggs with the induction of colorectal carcinoma-related signaling pathways in enterocytes based on co-culture experiments [54]. However, this interpretation of presented results may have not considered the whole picture, and it was later endorsed in the following review on schistosome-induced carcinogenesis [55]. Liver eggs have also been used to study the extravasation of eggs into intestinal tissues and the intestinal lumen. While the initial transition may be assumed to be similar for both localizations, drawn conclusions about the life cycle based solely on “losers” may not be entirely accurate [56]. The same oversight has been made by authors in recent literature attempting to elucidate the principles of egg migration in granulomas through the intestinal wall using liver-derived eggs [5]. Given our demonstration of significant differences between eggs from various organs, it is advisable to reevaluate previous conclusions drawn from results obtained using liver eggs. Moreover, inclusion of intestinal eggs in future studies could benefit the joint efforts to fill remaining knowledge gaps in schistosome egg biology and associated pathologies.

Finally, it must be taken into account that our organ comparison can be potentially burdened with bias due to the possible different age of mature eggs in the two tissues. While intestinal eggs leave the host after reaching maturity, mature liver-trapped eggs accumulate in the tissues. However, the chosen timepoint of 7 weeks post infection for eggs isolation should minimize the effect. Additionally, older eggs are encapsulated in large granulomas over time, and thanks to our chosen isolation method, these eggs remain on the isolation sieves and do not make it to the final analysis. Together with significant differences in the immature eggs we believe, that the observed differences in eggs based on the organ of origin are tissue and not age-specific and our analysis faithfully represent a snapshot of a true biological state at the chosen time point.

To sum up, here we present a comprehensive comparative gene expression study revealing cardinal differences between *S*. *mansoni* eggs isolated from the liver and intestine of experimentally infected mice. Our data not only show that tissue localization affects gene expression of the egg soon after oviposition and viability of the forming larva, but also that the host immune response differs depending on the specific antigenicity of the eggs in both tissues. These findings might indicate a significant two-way communication between the egg and the host. Key molecules identified in parasite-host interactions show clear preferences for gene expression depending on surrounding tissues, including the well-studied immunomodulators omega-1 and IPSE/alpha-1. Prioritizing the identification of tissue-specific factors responsible for changes in egg expression is essential as a follow-up task, along with delving deeper into the tissue-specific immune response in relation to *in situ* egg-secreted antigens. Finally, a quantitative proteomics assessment of SEA from liver and intestinal eggs could significantly expand our understanding of the observed phenomenon.

## 4 Materials and Methods

### 4.1 Ethics statement

Research involving experimental animals was performed in accordance with the animal welfare laws of Czechia and under the European regulations for transport, housing and care of laboratory animals (Directive 2010/63/EU amended by Regulation 2019/1010/EU). Experiments were approved by the Animal Welfare Committees of Charles University, University of Life Sciences and the Ministry of Education, Youth and Sports of Czechia (approval numbers MSMT-34303/2022-4 and MSMT-33097/2020-6). All animals used in the study were maintained by a certified person (Certificate Number CZ 02627) in an accredited facility (accreditation number 8615/2019-MZE-17214, both issued by the Ministry of Agriculture of the Czechia).

### 4.2 Collection of eggs

The life cycle of *S*. *mansoni* is maintained in our laboratories using *Biomphalaria glabrata* as an intermediate host snail and C57BL/6JRj (Janvier Labs) female mice as the definitive host. Mice were infected in a water bath using 150 cercariae per mouse for one hour. After 7 weeks of the infection, the mice were sacrificed, and their small intestines (emptied of contents and rinsed with PBS) and livers were used for egg isolation. The organs were mechanically homogenized using a benchtop blender and then incubated in 350 mM NaCl containing 1% trypsin from porcine pancreas (Sigma-Aldrich) at 37°C for 2 hours. Homogenates were then passed sequentially through 425 μm, 180 μm, 125 μm, and 45 μm mesh stainless steel sieves. Eggs were collected from the last sieve and further cleaned in Petri dishes using a swirling concentration technique.

The suspension of clean eggs was used for the preparation of SEA [57], or further separated as described in Ashton et al., 2001 [3]. As for the latter, the eggs were pipetted onto the top of 10 ml of 60% Percoll (Cytiva) mixed with 350 mM NaCl in a 15 ml tube. Eggs were separated by density gradient by centrifugation of Percoll on a swing rotor at 300g for 20 min at room temperature. The top 9 ml contained eggs that we refer to as *immature* and correspond to developmental stages 1–6 according to Jurberg et al. (2009) [4]. The bottom 1 ml contained eggs that we refer to as *mature* and correspond to developmental stages 7–8. Eggs were then washed from Percoll by repeated centrifugation in a 350 mM NaCl solution.

### 4.3 Viability assay

To evaluate the viability of hatched miracidia from liver- and intestine-derived eggs, we prepared a concentrated suspension of mature eggs. This suspension was then pipetted into 3 ml of unchlorinated spring water, and maintained at 25°C. The eggs were allowed to hatch over the course of one hour under artificial laboratory light. Following the hatching period, the newly hatched miracidia were transferred to fresh wells containing 200 μl of water and were immediately counted. The wells with miracidia were kept at 25°C under the artificial light source. After three hours, a recount was conducted to identify the miracidia that were still actively swimming or moving. The experiment was performed with 3 biological replicates, i.e., eggs isolated from 3 different mice.

### 4.4 RNA isolation, cDNA library preparation, and sequencing

Isolated and separated immature and mature eggs were incubated overnight in RNAlater Stabilization Solution (Thermo Fisher) and then stored at -80°C. For RNA isolation, the RNAlater Stabilization Solution was first replaced with Trizol, then the eggs were mechanically homogenized using a plastic pestle, and the total RNA was isolated according to the manufacturer’s instructions. RNA samples were treated with TURBO DNase (Invitrogen) according to the manufacturer’s manual. The concentration and purity of RNA were measured using NanoDrop Spectrophotometer (Thermo Scientific). Since it is difficult to isolate high-quality RNA from eggs, we chose a specific cDNA library preparation strategy, which is suitable for subsequent gene expression analysis even if the input RNA is partially degraded [58]. Therefore, 250 ng of total RNA per sample was used as input for library preparation using QuantSeq FWD 3’mRNA Library Prep Kit (Lexogen) in combination with UMI Second Strand Synthesis Module for QuantSeq FWD and Lexogen i5 6 nt Unique Dual Indexing Add-on Kit (Lexogen). Quality control for library quantity and size distribution was done using QuantiFluor dsDNA System (Promega) and High Sensitivity NGS Fragment Analysis Kit (Agilent Technologies). The final library pool was sequenced with NextSeq 500 (Illumina) using High Output Kit v2.5 75 Cycles (Illumina) in single-end mode, resulting in an average of 10 million reads per sample.

### 4.5 Differential gene expression analysis

Quality control of raw reads was performed in FastQC (v0.11.9). Reads were mapped to the *S*. *mansoni* genome (Wormbase ParaSite v9) [59–62] using STAR aligner (version 2.7.10a) [63] with options adjusted to the QuantSeq FWD 3’mRNA Library Prep Kit (Lexogen). The UMIs were deduplicated using open source UMI-tools software package (version 1.1.2) [64]. Deduplicated mapped reads were counted on the gene level using FeatureCounts (version 2.0.1) [65] with options -M and–fraction (counting of multi-mapped reads with expression value as a fraction based on the number of genes assigned, ranging from 2–20 genes). Gene length normalization was not performed, because it was not applicable to our sequencing strategy and could have biased the results if implemented. All genes from the genome were newly annotated with the name of the most similar protein from the UniProt database to make the results more descriptive and comprehensible. This was done using the pblast algorithm within the DIAMOND software (version 2.1.06) [66] against the Uniprot database (Unreviewed TrEMBL database) [67] the *S*. *mansoni* protein fasta (Wormbase Parasite v9) as a query with options for e-value >1e-50. Differential gene expression was analyzed and statistically evaluated using the DESeq2 R package (version 1.36.0) [68]. For PCA within DESeq2 package, log–transformed read count data was used as an input. Four comparisons of differential expression were performed: immature versus mature eggs from the liver, immature versus mature eggs from the intestine, immature eggs from liver versus those from intestine, mature eggs from liver versus those from intestine. Statistically significant differentially expressed genes were considered to be the ones with padj < 0.05 and log2 fold change > 1.

### 4.6 Functional enrichment analysis

Gene expression among the examined egg samples was further investigated using GO terms enrichment analysis to detect altered expression associated with groups of functional biological processes. Gene Ontology (GO) terms for S. mansoni genes were obtained using BioMart tool at WormBase Parasite (https://parasite.wormbase.org/) [61]. Enrichment analysis of differentially expressed genes (DEGs) identified with DESeq2 was performed using topGO v2.54.0 [69] with nodeSize = 5 and the weight 01 method. Raw pvalues were adjusted using False discovery rate (FDR) correction. GO terms with FDR < 0.05 were considered as significantly enriched.

### 4.7 Immunolocalization of IPSE/alpha-1 in mouse intestine and liver

Liver and small intestine from *S*. *mansoni* infected female mouse (C57BL/6JRj, Janvier) were collected 7 weeks post infection and rinsed in 1x PBS. Both tissues were fixed in 4% neutral buffered formalin for 3 days at the room temperature. All fixed samples were dehydrated with increasing concentrations of ethanol (50%, 70%, 90%, 96%, 100% v/v ethanol) for 20 min for each step. Subsequently, all tissues were cleared twice with xylene (VWR) for 15 min and embedded into the paraffin—Paraplast (Sigma-Aldrich). Sections (5 μm) were cut using microtome Shandon Finesse ME+ (Thermo Fisher Scientific) and placed onto X-tra adhesive slides (Leica).

Deparaffinized and rehydrated 5 μm mouse tissue sections (livers/intestines) were boiled for 3x 3 min in 0.05 M citrate buffer pH 6 containing 0.05% Tween 20 in a microwave oven (500 W) and then allowed to cool for 20 min. After that, the sections were blocked with a blocking buffer (PBS, 2% BSA, 0.25% Triton X-100, and Goat anti-mouse p(Ab) (Abcam, ab6668) (1:50) for 1 h. Finally, the sections were washed 3x in PBS-T (PBS, 0.25% Triton X-100) for 5 min followed by overnight incubation with monoclonal anti-IPSE/alpha-1 antibodies (kindly provided by Dr Gabriele Schramm) diluted 1:50 in antibody diluent (PBS, 1% BSA, 0.25% Triton X-100; 1:50 dilution) in the wet chamber at 4°C. The preparation of monoclonal antibodies was described in Schramm et al. (2003). For negative control, the healthy mouse serum was used. After incubation, the slides were washed 3x in PBS-T for 5 min and each slide was incubated with Goat Anti-Mouse IgG (H+L)—Alexa Fluor Plus 647 (Invitrogen, A48289) diluted 1:500 in PBS-T with 1% BSA for 1 h in the dark. Subsequently, slides were washed 3x in PBS-T for 5 min and mounted in Fluoroshield with DAPI (Sigma-Aldrich). Fluorescence signals were then excited and visualized by ZEISS Axio Scan Z1 Slide Scanner and confocal Leica TCS SP8 microscope. Lighting settings were selected using control slides probed with pre-immune serum to define the background signal threshold.

### 4.8 Cell isolation, stimulation and cytokine detection

Spleens and mesenteric lymph nodes (mLNs) were harvested from female mice (C57BL/6JRj, Janvier) 7 weeks post infection and uninfected, age-matched animals. Single-cell suspensions were prepared by passing the organs through 70 μm cell strainers. The splenocyte samples underwent red blood cell lysis by ACK (Ammonium-Chloride-Potassium) lysis buffer, as already described [70]. The cells wer​e resuspended in RPMI 1640 supplemented with 10% fetal bovine serum, 2 mmol/L l-glutamine, 100 U/mL penicillin, and 100 μg/mL streptomycin (all purchased from Merck). One million cells in 800 ul were seeded in 24-well plates, treated with liver or intestinal SEA (20 μg/ml), and cultivated for 72 hours (37°C, 5% CO_2_). Unstimulated and concanavalin A (1.25 μg/ml) treated cells served as negative and positive control, respectively. Concentrations of cytokines (IFN-γ, IL-4, IL-5, and IL-10) were then analyzed in undiluted cell culture supernatants by commercially available enzyme-linked immunosorbent assays (ELISA, BioLegend).

### 4.9 Detection of serum antibodies

SEA-specific serum antibodies were detected by homemade ELISA. Liver or intestinal SEA were diluted in carbonate buffer (1 ug/ml) and used for overnight coating of 96-well plates at 4°C. After washing with 0.05% Tween 20 in PBS, the wells were blocked with 1% BSA at 37°C for 2 hours. Then the wells were probed with diluted mouse serum (1:100) for 2 hours. After washing, the wells were incubated with horseradish peroxidase-conjugated goat anti-mouse IgG (1:8,000; Merck, #A2554) or IgG1 (1:2,500; Abcam, #ab97240) for 1.5 hours. Finally, the wells were repeatedly washed, and tetramethylbenzidine liquid substrate (Merck, #T4444) was used to visualize the reaction. The reaction was stopped by 1M HCl and read at 450 nm.

### 4.10 Statistical analysis

Quantitative data (other than the aforementioned gene expression) were analyzed in GraphPad Prism (v. 10). T-test or two-way analysis of variance followed by Holm-Šídák test were used, p-values <0.05 were considered significant.

## Supporting information

S1 Table Sequencing, mapping and counting statistics for each replicate. Abb.: INT = intestine; LIV = liver; im = immature egg; ma = mature egg.
(XLSX)

S2 Table Gene expression levels represented in Reads per Million of all sequenced replicates. Abb.: INT = intestine; LIV = liver; im = immature egg; ma = mature egg.
(XLSX)

S3 Table Differential expression analysis results (DESeq2). Four sheets list result for four pairwise comparisons. Sheet A compares intestinal immature and mature eggs. Sheet B compares liver immature and mature eggs. Sheet C compares immature intestinal and liver eggs. Sheet D compares mature intestinal and liver eggs. Last two columns of tables serve as filter for significantly upregulated genes (padj < 0.05; log2 fold change > 1). Abb.: INT = intestine; LIV = liver; im = immature egg; ma = mature egg.
(XLSX)

S4 Table Gene Ontology enrichment analysis results (topGO). (XLSX)

S5 Table Fig 4 genes of interest. (XLSX)

S6 Table Omega-1 & IPSE paralogues analysis. (XLSX)

S7 Table Minimal dataset for Fig 1B, 1C and Fig 6. (XLSX)

S1 Fig Comparative gene expression analysis, investigating mitochondrial genes (A) and genes encoding molecules with potential roles in egg-host interaction (B, C, D) within immature *S*. *mansoni* eggs obtained from the intestine and liver of experimentally infected mice. Volcano plots show genes that were statistically significantly differentially expressed (DESeq2 analysis; padj < 0.05; log2 fold change > 1). This encompasses (A) genes encoded in mitochondrial genome, (B) micro-exon genes (MEGs) and venom allergen-like proteins (VALs), (C) immunomodulatory molecules IPSE/alpha-1 and omega-1 and (D) proteases and inhibitors in *S*. *mansoni* eggs. The corresponding heatmaps show a mean of gene expression levels normalized to Reads per Million (RPM), serving to highlight the proportional contributions of each subgroup to the overall gene expression profile in a given sample.
(PNG)

S2 Fig Negative control for immunolocalization of IPSE/alpha-1 in mouse intestine (A) and liver (B) sections infected with *S*. *mansoni*. Labels are represented by: red for anti-IPSE/alpha-1 antibody-specific sites, green for eggshell autofluorescence, and blue for DAPI-labeled cell nuclei. Scale bar = 20 μm.
(PNG)

S3 Fig **(A) The ratio of immature to mature *S*. *mansoni* eggs isolated from the liver and intestine of mice.** The graph shows the average egg ratios obtained from 4 mice after separation via Percoll gradient. The error bars represent the standard deviation. (B) The actual eggs counts from which the ratios were calculated. Data were evaluated by 2-way ANOVA followed by Šídák’s multiple comparisons test.
(PNG)
