# Supplementary figures and images for: Winners vs. losers: Schistosoma mansoni intestinal and liver eggs exhibit striking differences in gene expression and immunogenicity
Source: PLoS Pathog. 2024 May 30;20(5):e1012268. doi: 10.1371/journal.ppat.1012268 (PMC11166329; doi:10.1371/journal.ppat.1012268)

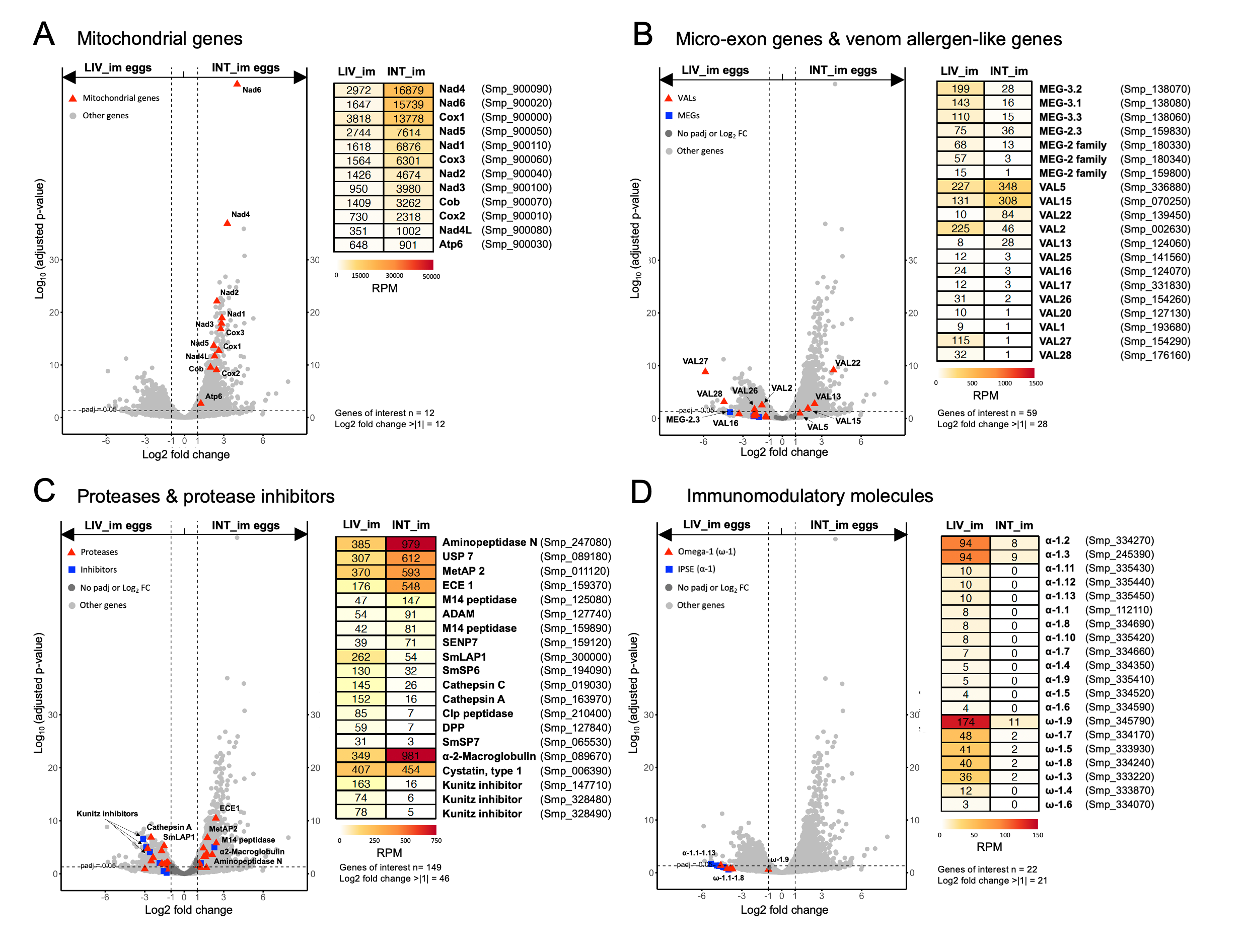

Supplement: S1 Fig — Volcano plots show genes that were statistically significantly differentially expressed (DESeq2 analysis; padj < 0.05; log2 fold change > 1). This encompasses (A) genes encoded in mitochondrial genome, (B) micro-exon genes (MEGs) and venom allergen-like proteins (VALs), (C) immunomodulatory molecules IPSE/alpha-1 and omega-1 and (D) proteases and inhibitors in S. mansoni eggs. The corresponding heatmaps show a mean of gene expression levels normalized to Reads per Million (RPM), serving to highlight the proportional contributions of each subgroup to the overall gene expression profile in a given sample. (PNG) [file ppat.1012268.s008.png]

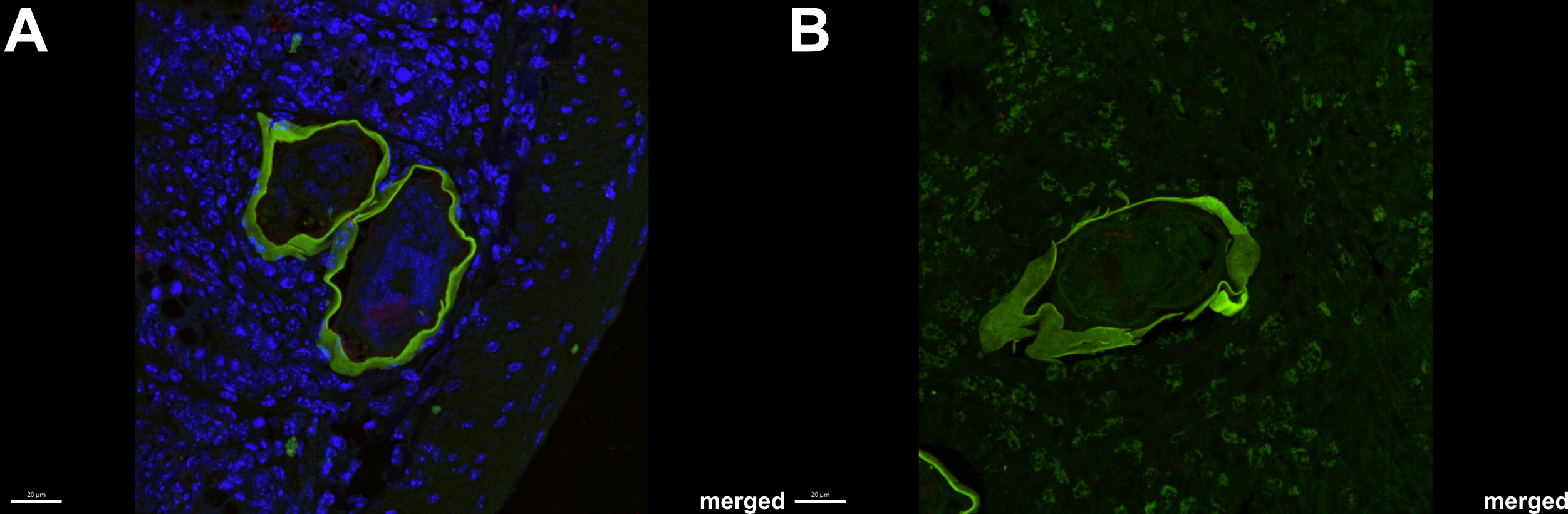

Supplement: S2 Fig — Labels are represented by: red for anti-IPSE/alpha-1 antibody-specific sites, green for eggshell autofluorescence, and blue for DAPI-labeled cell nuclei. Scale bar = 20 μm. (PNG) [file ppat.1012268.s009.png]

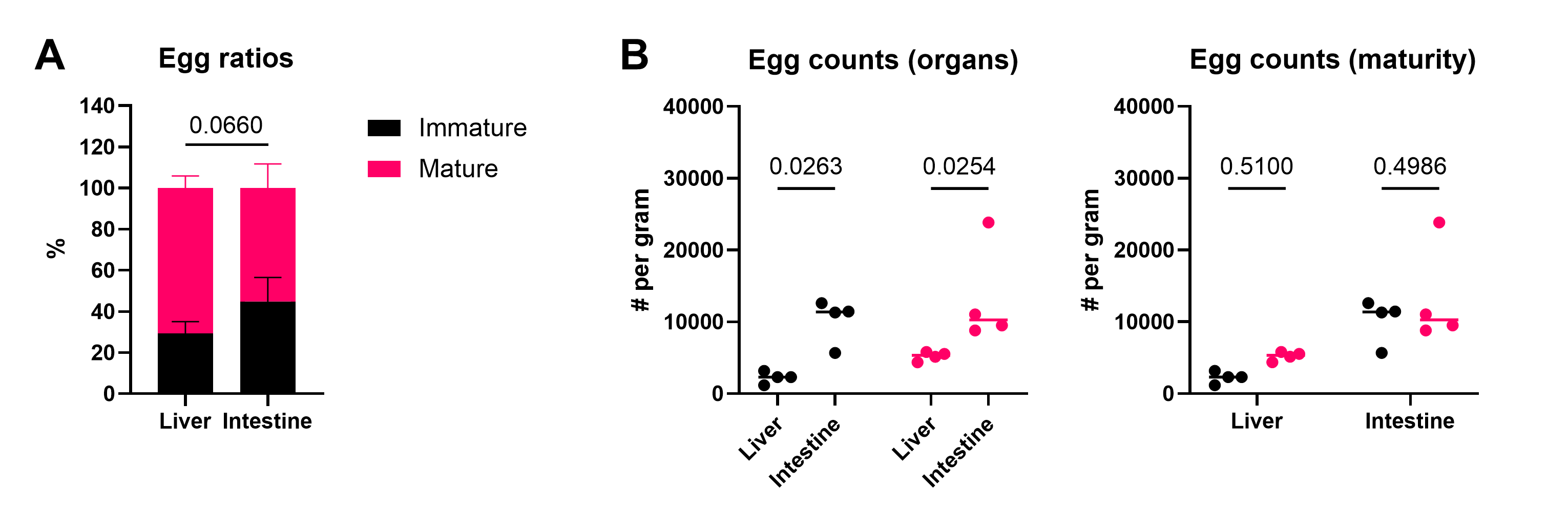

Supplement: S3 Fig — (A) The ratio of immature to mature S. mansoni eggs isolated from the liver and intestine of mice. The graph shows the average egg ratios obtained from 4 mice after separation via Percoll gradient. The error bars represent the standard deviation. (B) The actual eggs counts from which the ratios were calculated. Data were evaluated by 2-way ANOVA followed by Šídák’s multiple comparisons test. (PNG) [file ppat.1012268.s010.png]
